# Conceptual thermal constraints on the growth of the first tree on a terraformed Mars

**DOI:** 10.1371/journal.pone.0349588

**Published:** 2026-06-12

**Authors:** Agnieszka Wendland, Piotr Pałka, Robert Olszewski, Alison F.C. Bridger, Melinda A. Kahre, Christian Körner, Christopher P. McKay

**Affiliations:** 1 Warsaw University of Technology, Warsaw, Poland; 2 San Jose State University, San Jose, California, United States of America; 3 NASA Ames Research Center, Moffett Field, California, United States of America; 4 University of Basel, Basel, Switzerland; National Autonomous University of Mexico Institute of Geophysics: Universidad Nacional Autonoma de Mexico Instituto de Geofisica, MEXICO

## Abstract

The environmental conditions on present‑day Mars are far outside the range tolerated by known complex terrestrial life. Conceptual climate studies have suggested that, in hypothetical terraforming scenarios, artificially enhancing the greenhouse effect could restore Mars to more habitable surface conditions. Early colonizing terrestrial life on a warming Mars would plausibly consist of lichens and high‑alpine or high‑arctic plants. Here, we consider a later, more demanding step and investigate the thermal conditions under which the first tree could, in principle, grow on the Martian surface. Based on empirical treeline studies, we adopt thermal thresholds for a representative high‑elevation conifer: a growing season of at least 110 sols during which daily minimum temperatures exceed −6 °C, daily means exceed 6 °C, and daily maxima remain below 40 °C. In addition to liquid water and suitable substrates, O₂ at ~1 hPa and non‑toxic CO₂ levels are likely required; however, these non‑thermal constraints are not explicitly modelled and make the temperature thresholds necessary but not sufficient for tree viability. We use a high‑resolution surface energy balance model of Mars, assuming a pure CO₂ atmosphere with prescribed grey infrared opacity and neglecting the coupled water cycle, full atmospheric dynamics, photochemistry, and surface radiation, to estimate spatio‑temporal thermal windows for potential tree growth as a function of CO₂ surface pressure and additional greenhouse forcing. For a 100 hPa CO₂ atmosphere, near‑surface temperatures satisfying the treeline thresholds first appear when the added grey infrared opacity is ~ 0.39 optical depths. In our simulations, these thermal criteria are initially met not in the tropics (±25°), but in the low‑lying Hellas Basin. As either the CO₂ surface pressure or the imposed grey opacity is increased beyond the values required to open the thermal window, large regions of the southern hemisphere subsequently become thermally overheated and thus unsuitable for tree growth. In this sense, the thermal windows identified in our simulations mark conditions under which temperature would no longer be the primary limiting factor for tree growth, assuming that other essential environmental constraints (such as water availability, radiation environment, substrate properties, and atmospheric composition) are satisfied. We emphasize that this study deals with temperature only, which is an important factor in tree growth on Mars. Other factors that affect tree growth, including water, CO₂ limits, O_2_ limit, UV and ionizing radiation, and soil nutrients and microbial population, are not considered explicitly here.

## Introduction

Mars today is not suitable for life as we know it and for sustained surface habitability due to the low pressure of its atmosphere (the pressure is ~ 6 hPa composed of 95% CO₂ with the balance dominated by N_2_ and Ar and only 0.13% O_2_ and the resulting low temperature of its surface (mean ~ –60°C), together producing a lack of liquid water [1,2]. One of the most interesting discoveries in planetary science is that Mars was habitable for life over an extended period in its early history (e.g., [2,3]). Lovelock and Allaby [4] suggested, in a conceptual framework, that artificially enhancing the greenhouse effect on Mars could, in principle, restore more Earth‑like surface conditions – a process referred to as “terraforming” [5]. Further studies have developed this idea based on the introduction of greenhouse gases [6–9], artificial control of solar lamination [10], and the use of nanoparticles [11]. These studies suggest that thermodynamically warming Mars is feasible on timescales of hundreds of years. However, a fundamental uncertainty is the amount of CO₂, H₂O and N₂ on Mars [7,12]. Recent work has further explored the climatic and volatile constraints on a warm, thick‑atmosphere Mars, including detailed modelling of CO₂–H₂ greenhouse scenarios for early Mars [13], reassessments of how much CO₂ could realistically be mobilized from the regolith into the atmosphere [12], and comprehensive reviews of early Martian climate that evaluate the plausibility of various warming mechanisms [14,15].

Unfortunately, our understanding of the global inventory of the key volatile building blocks of remains limited. Recent rover missions, such as Mars Science Laboratory, have greatly improved constraints on the composition of Martian soils and rocks, including nutrients and redox‑sensitive species relevant for habitability (e.g., [16,17]), but they still provide only indirect constraints on the subsurface reservoirs of CO₂, H₂O and N‑bearing phases. The key climate‑engineering question for warming Mars remains the total amount of CO₂ that can be mobilized into the atmosphere [12]. To be conservative, we adopt a pessimistic estimate of the accessible CO₂ inventory corresponding to a surface pressure of ~100 hPa [18]. Throughout this paper we therefore treat 100 hPa of CO₂ as a pessimistic but physically plausible upper bound on atmospheres attainable with currently recognized CO₂ inventories, and regard the 500–1000 hPa cases as illustrative high‑pressure extensions used only to explore how the thermal window behaves at much larger total pressures. As discussed below, plants – and by extension trees – have been shown in laboratory experiments to grow at total pressures of order 100 hPa, although not in pure CO₂ and with a finite partial pressure of O₂. [19]

The hypothetical feasibility of human settlements and terraforming Mars has motivated a considerable literature on the associated environmental ethics [20–25] expressing positive views based largely on the intrinsic worth of life and negative views based on preservation of natural systems.

While several studies have addressed biological aspects of terraforming and in‑situ resource use, relatively fewer have quantified specific physiological thresholds for complex terrestrial organisms under modified Martian climates. In a seminal work, [26] proposed a conceptual model for ecological succession based on an analogy with descent down a terrestrial mountain. Each drop in elevation results in a warmer, wetter climate and a more diverse biological community. Following this metaphor, it is plausible that early colonizing organisms on a warming Mars would resemble lichens and high‑alpine or high‑arctic plants that tolerate extreme temperature regimes [27].

Experimental and conceptual studies of terrestrial organisms under Mars like conditions highlight both the potential and the limitations of biological involvement in terraforming scenarios. Lichen exposure experiments have shown that certain Antarctic species can physiologically adapt to simulated Martian surface conditions on relatively short timescales [27], suggesting that some extremophiles may tolerate aspects of the present day Martian environment. At the same time, research on higher plants in controlled environments and Mars analog substrates demonstrates that plant growth is possible but tightly constrained by factors such as substrate composition, atmospheric pressure, gas mixture, and radiation [28–30]. [31] experimentally tested the growth of Antarctic plant species on Martian and Lunar soil simulants under terrestrial atmospheric conditions, showing that at least some extremophile plants can germinate and develop on regolith analogs. These results support the idea that cold-adapted terrestrial flora could be promising candidates for early biological experiments and bioregenerative concepts in Mars and Moon exploration and, in a broader sense, for long‑term terraforming scenarios. Together, these studies motivate quantitative assessments of the physical and chemical boundary conditions - - such as those explored here – that would be required for more complex terrestrial biota to contribute meaningfully to a terraforming process on Mars.

One of the most striking features of mountain ecosystems is the treeline – a typically sharp discontinuity above which trees do not grow. Trees are highly efficient producers of biomass and, if their biomass is partially sequestered, they could contribute significantly to atmospheric O₂ accumulation in terraforming scenarios. This motivates a quantitative assessment of how and where trees could, in principle, grow on a warming Mars. We assume that the necessary microbial community to support tree growth is part of the inoculum added to the soil with the seeds of the trees. The role of plants in terraforming Mars remains of interest; recently, [32] discussed utilizing algae as biocatalysts for making Mars habitable.

In this paper, we use a high-resolution simulation of the Martian surface as it is warmed by the artificial enhancement of the greenhouse effect to determine when and where trees could grow on Mars. In this paper, we focus on surface temperature as a first‑order environmental variable that is directly affected by increases in atmospheric pressure and greenhouse forcing, and that controls the CO₂ cycle and the potential formation of liquid water. Other key factors for plant viability on Mars – including radiation, regolith chemistry, nutrient availability, and a functional microbiome – are not explicitly modelled here and remain essential topics for future work^.^ We also consider how increases in CO₂ in the Martian atmosphere would increase the O2 partial pressure to the level required for plant life, but on the other hand CO₂ is toxic to plants above a certain level. There are many other caveats to note in such a temperature focused exercise. For instance, we assume a functional microbiome in the substrate to come with the first trees, gradually yielding soil, ensuring litter recycling, nutrient provision etc. We also acknowledge that moisture, both in the substrate and in the atmosphere, must match fundamental requirements.

Our approach deliberately includes only a subset of physical factors – primarily pressure and thermal conditions – and does not attempt to model the full set of biological requirements necessary for tree survival and growth. The purpose of this model is to estimate the spatio‑temporal “thermal window” for potential tree growth on Mars as a function of CO₂ pressure and additional greenhouse forcing, rather than to provide a comprehensive assessment of the practical feasibility of terraforming.

### Temperatures, pressure, oxygen, and water for trees

Over the past few decades, two important observations have informed our understanding of the elevation of the treeline. First, the treeline, properly so-called, is the fundamental limit of the growth of trees [33,34]. Second, numerous observations indicate that the limit of growth of trees at high elevations is correlated with temperatures during the growing seasons, and is surprisingly independent of the speci which is linked to the fact that trees stand up above the surface boundary layer and are exposed to the atmosphere. A model organism for the first tree on Mars could be the Siberian pine (*Pinus sibirica*), a close relative, if not synonymous, with *Pinus cembra,* a widely studied species in Europe. We adopt this species as a proxy for a representative high‑elevation conifer because it is widespread, well‑characterized, and highly tolerant of harsh atmospheric conditions and low temperatures. Our intention is to use a typical coniferous tree as an illustrative example in the model, while recognizing that the results are sensitive to the chosen thermal thresholds. This choice is therefore part of a conceptual exercise aimed at highlighting the potential possibilities and limitations of transferring terrestrial life to the Martian surface. Based on [35,36], we set the temperature limits for trees on Mars to be: (i) the growing season must last at least 110 sols (Martian days) during which: (ii) the minimum temperature during the growing season must be > −6 °C, (iii) the average daily temperature must be > 6 °C, and finally (iv) the maximum temperature must be < 40°C based on observations averaged across multiple high‑elevation treeline sites (data from [37–39]).

These treeline thresholds are derived from terrestrial high‑elevation and high‑latitude sites that benefit from active hydrological cycles, well‑developed soils, and oxygen‑rich atmospheres. In this study we use them strictly as proxy thermal limits for a representative high‑elevation conifer (Pinus sibirica/ cembra) and adopt their direct application to Mars as a simplifying assumption within a conceptual framework. In Martian conditions, the effective thresholds for growth might need to be shifted due to differences in atmospheric composition, radiation environment, gravity, and soil development, and our results should therefore be interpreted as indicative rather than as precise quantitative predictions for any particular species.

The treeline‐based temperature thresholds adopted in this study should be understood as **necessary but not sufficient conditions** for tree growth. Our four criteria (minimum growing season length, thresholds for daily minimum and mean temperature, and an upper limit on daily maxima) specify a thermal window within which trees could, in principle, function, but they do not guarantee viability in the absence of other key environmental requirements. In particular, our model does not include explicit constraints on water availability or the hydrological cycle, the surface radiation environment, regolith properties and soil development, or the presence of a functional microbial community supporting nutrient cycling. By design, the simulations isolate thermal constraints only; satisfying the temperature thresholds identified here would therefore have to be complemented by adequate water, suitable substrates, radiation protection, and a supporting microbiome before real trees could persist on Mars.

This is also the case on Earth, the fundamental limit of the growth of trees sets the treeline elevation but tree growth on Earth often does not reach this limit due to a variety of local factors and even human activities [34].

Design studies of greenhouses on Mars and the Moon have motivated extensive experiments with growing plants, although not large trees, at reduced atmospheric pressure (as low as 100 hPa) and with a high fraction of CO₂ and reduced O₂ (e.g., [40–43]). More recent experimental and conceptual studies of controlled‑environment agriculture and bioregenerative life support for space missions further explore plant performance under reduced pressure and Martian‑analog conditions [28–30]. For plant growth at a total pressure of 100 hPa the optimal CO₂ level is ~ 1 [41–43] and levels above ~ 10 hPa would be toxic [44,45]. This optimal atmospheric composition would imply 99 hPa of N_2_ in the atmosphere of Mars presumably from decomposition of the soil nitrates [46,47], which are known to be present, but the total inventory is undetermined. In our calculations of the temperature, we assume pure CO₂. This only slightly overestimates the warming effect because the greenhouse warming of 100 hPa is small and the major part of the warming is due to the artificial infrared opacity.

Plants need O_2_ for growth, and hypoxia is typically reported in plants when O₂ levels fall to 1–5% (e.g., [48,49]). For example, [43] reported in radishes that O_2_ as low as 70 hPa had no deleterious effects, but a significant reduction in growth was observed when the oxygen partial pressure was dropped to 20 hPa, regardless of the total pressure. However, the mitochondrial enzyme that requires oxygen has such a strong affinity that this enzyme can function with oxygen partial pressures of 0.1 hPa [50] suggesting directed adaptation to lower oxygen levels may be possible. Notwithstanding this point, we take 1 hPa as the working threshold for O₂ levels required for tree growth, based on previous conceptual analyses and physiological considerations.

Recent observations and modelling studies place strong constraints on the present and past climate of Mars, as well as on plausible atmospheric evolution pathways relevant to terraforming scenarios. In situ measurements by the Curiosity rover have refined our knowledge of the current Martian atmospheric composition and isotopic ratios, revealing a thin, CO₂ dominated atmosphere with signatures of substantial past loss [51,52]. Climate and habitability studies of early Mars highlight both the difficulty of sustaining warm, wet surface conditions and the sensitivity of the water cycle and atmospheric stability to pressure and radiative forcing [53]. In parallel, exoplanet climate–photochemistry models of dense CO₂‑rich atmospheres provide analogues for how greenhouse gases and secondary species such as O₂ might accumulate or be depleted under different stellar and climatic regimes [54,55]. Together, these lines of evidence underscore that any terraforming scale modification of the Martian atmosphere must contend with tight constraints on volatile inventories, escape processes, and the coupled radiative–chemical response of a CO₂ dominated climate system.

It is important to note that the atmospheric states used in our thermal simulations – pure CO₂ at total pressures between 10 and 1000 hPa - are not self‑consistent with the combination of plant physiological constraints discussed above, namely an optimal CO₂ partial pressure of order ~1 hPa, an upper limit of ~10 hPa for non‑toxic CO₂, and O₂ partial pressures of ~1 hPa for respiration. In our framework, these pure‑CO₂ scenarios should therefore be viewed as a parameter sweep for surface temperature as a function of total pressure and grey infrared opacity, rather than as realistic terraforming end‑states. Achieving all three requirements simultaneously (sufficient total pressure, non‑toxic CO₂, and adequate O₂) would likely demand a complex combination of processes, including mobilization of CO₂, addition of a background gas such as N₂, and active engineering of the atmospheric composition, none of which are modelled here.

On Mars today, O₂ is a minor constituent of the near‑surface atmosphere, with a volume mixing ratio of order 0.1% and corresponding partial pressures of a few 10 ⁻ ³–10 ⁻ ² hPa under present conditions, as constrained by in situ measurements and photochemical modelling of modern Martian atmosphere (e.g., [51,52,56]). The question of interest here is how the O_2_ fraction will scale as the CO₂ content of the atmosphere is increased from the present 6 hPa to 100 hPa. If the fraction of O_2_ remains constant at 0.13%, then there is not enough O_2_ since 0.13% of 100 hPa = 0.13 hPa – well below the level of O_2_ needed.

The O_2_ concentration in CO₂-dominated atmospheres has been considered in studies of early Mars and in considerations of spectroscopic detection of O_2_ as a sign of life on exoplanets. [57] considered O_2_ in a CO₂-dominated atmosphere on Mars, and obtained 1hPa of O_2_ for a 1000 hPa CO₂ atmosphere. The O_2_ dropped to very low levels as CO₂ decreased, becoming less than 0.1 hPa at 100 hPa of CO₂. [58] explicitly considered the O_2_ levels in a terraformed Mars, assuming a pressure of CO₂ of 1000 hPa. They found between 0.5 hPa and 20 hPa of O_2_ depending on the thermal profile in the stratosphere. The higher O_2_ values corresponded to a cold stratosphere with low levels of water (see Table 3 of [58]). Segura et al. [59] criticized the applicability of the [58] results for exoplanets, arguing “that abiotic O_2_ buildup is only likely to occur on dry or frozen planets”. This is perhaps not applicable to Earth but would perhaps pertain to Mars. Selsis et al. [58] did not present results for a 100 hPa CO₂ atmosphere but if the mixing ratio persisted down to this level the result would be 2 hPa O_2_ in a 100 hPa CO₂ atmosphere -much higher than the results reported in [57]. Recent coupled climate–photochemistry studies of CO₂‑dominated atmospheres on Mars and terrestrial exoplanets provide additional context for our assumptions about oxygen production and retention. For example, [60] modelled the early Martian climate under a denser CO₂ atmosphere and highlighted the strong coupling between atmospheric pressure, temperature, and the water cycle. [54,55] explored the photochemical and radiative behavior of high‑CO₂ atmospheres in the context of exoplanet biosignatures, showing that substantial abiotic O₂ can accumulate under certain climatic and stellar conditions. These studies underscore that O₂ abundances in CO₂‑rich atmospheres are highly sensitive to the thermal structure and water availability, and therefore remain a major uncertainty in assessing the viability of complex plant life on a terraformed Mars.

Thus, we consider that the O_2_ level on Mars in a 100 hPa CO₂ atmosphere is not certain, and more work needs to be done including models that limit the CO₂ to <10 hPa with the total pressure set by N_2_. However, current models suggest that at least initially when Mars is still cold and dry over most of the planet, O_2_ mixing ratios due to photochemical production may be high enough to support the first tree. If not, then increasing the pressure of CO₂ to as high as 500 hPa may be necessary. This would exceed the currently recognized sources of CO₂ on Mars and would also be far above the 10 hPa maximum limit of CO₂ pressure for plant growth.

The discussion of potential O₂ accumulation in terraforming scenarios should be regarded as highly uncertain. Existing photochemical models of CO₂‑dominated atmospheres on Mars and terrestrial exoplanets yield a wide range of possible O₂ mixing ratios, depending sensitively on the thermal structure, water abundance, and escape processes, and thus cannot yet be treated as firm predictions for a terraformed Mars. In this context, our analysis does not rely on any specific O₂ value: the thermal windows we identify are independent of the actual O₂ abundance and merely indicate where temperature and pressure conditions would not preclude tree growth. We refer to O₂ only as an additional constraint that would have to be satisfied, alongside limitations on CO₂ toxicity and total pressure, for real trees to survive, and we do not imply that achieving favorable O₂ levels is likely or guaranteed.

Comprehensive GCM studies of the Martian water cycle under a significantly thicker atmosphere, while assuming the present‐day obliquity and solar luminosity, remain limited. However there have been simulations of the water cycle in a higher pressure atmosphere on early Mars, with a reduced solar luminosity (e.g., [60,61]). These indicate that a thicker atmosphere would transport water from the poles to equatorial regions more effectively. These models show that increasing atmospheric pressure generally enhances the efficiency of water transport away from the poles, but stable surface liquid water remains difficult to achieve without additional warming [14,53]. However, in these models with reduced solar luminosity, the water that accumulates on the surface remains frozen. Similar results are seen in models of the water cycle of Mars at high obliquity (e.g., [62,63]), including studies with our baseline model [64].Even with the present solar luminosity conditions, Mars remains too cold for this moisture to be a significant source of liquid water [64]. However, these models of early Mars and Mars at high obliquity indicate that as the atmospheric pressure increases, there is more effective transport of water from the polar regions to mid latitudes. In considering the first tree on Mars, we make a qualitative inference that a higher‑pressure atmosphere would lead to warmer conditions and, in turn, to more effective transport of water toward mid‑latitudes and an increased likelihood of transient liquid water, thereby improving the potential for tree growth. However, this remains a conceptual assumption rather than a result of explicit hydrological modelling, and a fully coupled simulation of the Martian water cycle under thick‑atmosphere scenarios is an important avenue for future research.

In our thermal simulations the atmosphere is treated as pure CO₂, with total surface pressures ranging from 10 to 1000 hPa and an externally prescribed grey infrared opacity. This choice is intended as a computational device to span a range of thermal states, rather than as a self‑consistent representation of atmospheres compatible with plant physiology. Laboratory and greenhouse studies indicate that for higher plants the optimal CO₂ partial pressure is of order ~1 hPa, with growth inhibition and toxicity becoming significant above ~10 hPa. Thus, the pure‑CO₂ atmospheres considered here are not directly compatible with these biological thresholds. In any realistic scenario where terrestrial trees could grow on Mars, most of the total pressure would instead have to be provided by an inert background gas such as N₂, with CO₂ restricted to the physiologically acceptable range and O₂ maintained near ~1 hPa for respiration. Under such conditions, similar total pressures and effective longwave opacities could, in principle, be achieved with much lower CO₂ fractions, and our simulations should therefore be interpreted as mapping thermal states as a function of total pressure and τ, not as literal plant‑ready atmospheric compositions.

In addition to temperature and pressure, the surface radiation environment on Mars represents an important independent constraint on complex plant life. Even under low‑pressure or moderately thick CO₂ atmospheres, ultraviolet and ionizing radiation at the Martian surface are expected to remain substantially higher than on present‑day Earth due to the lack of a global magnetosphere and ozone layer. Our model does not attempt to quantify these radiative effects or their biological impact, and they therefore lie outside the scope of the present thermal analysis. Nevertheless, any realistic assessment of the viability of trees or forests on Mars would need to consider radiation mitigation measures – such as partial burial in soil, local topographic shielding, or engineered atmospheric composition – in addition to satisfying the thermal thresholds discussed here.

### Surface energy balance model assumptions and limitations

We have developed an energy balance model for Mars [64]. The planet is discretized into 4002 equal-area polygons (3990 hexagons and 12 pentagons; area ≈ 36,000 km² each), providing uniform horizontal resolution and avoiding polar singularities. Vertical structure is treated in bulk: a surface–subsurface layer in thermal contact with the atmosphere, without explicit vertical levels.

In this study, we employ a simplified but spatially explicit surface energy balance model of Mars. The atmosphere is treated as composed of pure CO₂ for the thermal calculations, so that the prescribed CO₂ surface pressure directly sets the background greenhouse contribution; this choice slightly overestimates warming relative to mixed N₂–CO₂ atmospheres at the same total pressure. Additionally, artificial greenhouse forcing is represented by a single grey infrared opacity parameter, which we vary externally rather than computing from detailed radiative transfer or aerosol microphysics. We treat the grey infrared opacity τ as an effective, vertically integrated longwave optical depth that represents the combined impact of additional greenhouse absorbers (for example, engineered gases, aerosols, or enhanced CO₂ columns) on the outgoing thermal radiation. The grey infrared opacity τ is a mathematical construct: it is defined as the value of a uniform grey absorber across the thermal infrared spectrum that yields the same downward infrared flux as obtained from detailed line‑by‑line radiative transfer calculations (e.g., [9]). In this study, τ is therefore used as an abstract sensitivity parameter that controls the strength of the generic greenhouse effect, rather than as a directly measurable physical quantity tied to any specific gas or aerosol species. Increasing τ in our model reduces the effective infrared cooling to space and therefore raises the surface temperature, analogous to an increase in radiative forcing in more detailed radiative–convective or 3‑D GCM studies. This parametrization is intentionally simple and is used to explore the sensitivity of the thermal field to a generic increase in greenhouse effect, rather than to represent any specific engineered greenhouse species.

In this framework, we explicitly neglect several processes that are known to be important for the Martian climate system. In particular, the model does not resolve the hydrological cycle of H₂O, including cloud formation, atmospheric moisture transport, and phase changes of water, nor does it contain a full three‑dimensional dynamical core capable of representing baroclinic waves, transient eddies, and detailed Hadley circulation structure. As a result, our simulations do not address the stability or spatial distribution of surface or near‑surface liquid water, nor do they capture feedbacks involving water clouds or water vapor. The model should therefore be regarded as a first‑order thermal framework that isolates the radiative–conductive response of the surface–atmosphere system under prescribed CO₂ pressures and infrared opacities, rather than as a full general circulation model suitable for comprehensive climate or hydrological predictions.

The model does not include a fully coupled water cycle: water vapor, clouds, and phase changes of H₂O are not prognostic variables, and any effects of water on radiative transfer or latent heat are therefore neglected. Large‑scale atmospheric circulation and lateral heat transport are represented in a parameterized form using eddy diffusion between neighboring cells and a zonally averaged Hadley‑cell‑like heat flux calibrated against outputs from modern Mars GCMs, rather than being resolved explicitly by a full three‑dimensional dynamical core. Our framework is therefore not a full three‑dimensional general circulation model: it does not explicitly resolve atmospheric dynamics, cloud and dust microphysics, or photochemical processes, and should be viewed as a first‑order thermal model that isolates the radiative–conductive response of the surface–atmosphere system under prescribed CO₂ pressures and infrared opacities.

In the thermal calculations presented here we treat the Martian atmosphere as composed of pure CO₂. This simplification is motivated by the fact that, at the relatively low surface pressures considered (~100 hPa), the direct greenhouse contribution of CO₂ alone is modest and most of the additional warming in our scenarios is produced by the prescribed grey infrared opacity. Replacing part of the CO₂ with radiatively inert N₂ or with O₂ at fixed total pressure would slightly reduce the baseline CO₂ greenhouse effect but would leave the imposed grey forcing unchanged. Consequently, within the conceptual framework of this study, the location and extent of the “thermal windows” identified for potential tree growth are only weakly sensitive to the detailed atmospheric mixture at a given total pressure and τ. A more realistic treatment of N₂–CO₂–O₂ mixtures, and of specific engineered greenhouse species, could be achieved in future work by coupling our surface energy balance model to more detailed radiative transfer calculations.

In our model, the temperature of a cell *n* on sol *t* + 1, $\overset{t+\text{1}}{\underset{n}{Ts}}$*,* is determined from the temperature on sol *t*, $T\overset{t}{\underset{n}{s}}$*,* by an energy balance calculation:


$$\begin{array}{c} \frac{cM_{n}}{\Delta t}\left(T\overset{t+\text{1}}{\underset{n}{s}}-T\overset{t}{\underset{n}{s}}\right)=(\text{1}-A)(\text{1}-R){\overset{t}{\underset{s}{F}}}_{n}\left(\theta_{n},L_{s}\right)\;+\;\overset{t}{\underset{g_{n}}{F}}-\varepsilon \sigma {\left(T\overset{t}{\underset{n}{s}}\right)}^{\text{4}}+\Delta \overset{t}{\underset{n}{C}}+\Delta \overset{t}{\underset{n}{L}}+\Delta \overset{t}{\underset{n}{E}}-\Delta \overset{t}{\underset{n}{G}} \end{array}$$ (1)

Where $M_{n}$is the mass per unit area of the subsurface and lower atmosphere of the cell n, c is the specific heat per unit mass, $\overset{t}{\underset{s_{n}}{F}}(\theta_{n},L_{s})\;$is the solar flux which is a function of latitude, $\theta_{n}$ of the cell n*,* and the time of year and specific time of a day *h*, and depends on the orbital parameters of Mars especially the obliquity. A is the albedo of the cell, R is a reduction in solar flux due to dust, which depends on latitude and season. $\overset{t}{\underset{g_{n}}{F}}$ is downward greenhouse flux in cell n, *ε* is the emissivity of the surface, *σ* is the Stefan Boltzman constant, $\Delta \overset{t}{\underset{n}{C}}$ is the net convective heating carried into the cell from adjacent cells by eddy convection accounting for elevation differences in cells. $\Delta \overset{t}{\underset{n}{L}}$ is the latent heat in the cell n, released due to the condensation of atmospheric CO₂ or removed due to the evaporation of solid CO₂ in the cell. $\Delta \overset{t}{\underset{n}{E}}$ is the heat flow into the cell from the large scale circulation. $\Delta \overset{t}{\underset{n}{G}}$ is the downward flux to the subsurface ground layer. All fluxes on the right hand side of Eq. 1 are per unit area per unit time. Δt is a time step of simulation, equal to one sol (88,775 sec.) for the baseline, sol-averaged, model and half a Martian hour (1/48 sol, approx. 1850 sec.) for the diurnal model which resolves the sol in 48 timesteps. To compute the greenhouse flux, we assume an atmospheric temperature profile that is in radiative equilibrium, that connects the surface temperature to the effective temperature at the top of the atmosphere, and a gray infrared opacity. The total exchangeable CO₂ is set as a model parameter. This amount, less any CO₂ condensate, determines the mean pressure of CO₂ on the planet. The CO₂ pressure in an individual cell is set with respect to this mean assuming hydrostatic equilibrium. To reproduce the observed asymmetry in seasonal CO₂ cap persistence between the poles, we introduce a dust-related shading parameter R that reduces absorbed solar flux more strongly over selected high-latitude cells. The model is computationally efficient (one Martian year in <10 minutes on a standard desktop), enabling multi-decadal integrations and parameter sweeps. Baseline simulations are evaluated against Viking 1 and 2 lander records and two 3-D GCMs (MCD and the NASA Ames model v3.1). Although our model omits explicit vertical resolution, its high and uniform horizontal resolution yields global-mean surface temperatures within ~2–3 K of the GCMs (with the baseline and MCD solutions ≈2.3 K warmer than the NASA model).

The model provides a horizontally high-resolution, energetically consistent framework for simulating the coupled evolution of surface temperature, CO₂ frost, and pressure on Mars under prescribed orbital, radiative, and dust conditions, suitable both for baseline present-day climate and sensitivity experiments.

**Fig 1:**
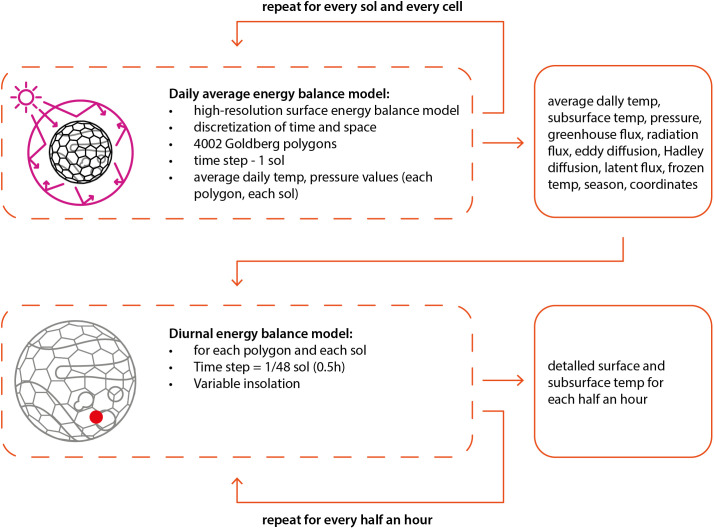
Diagram of the calculation process.

Compared to the baseline model [64], the model described in this article has been extended in two ways (Fig 1):

- The temporal resolution was increased by an order of magnitude (computational step of 1/48 sol compared to 1 sol in the baseline model), the spatial resolution remained the same (4002 cells – Goldberg polygons). This was done to resolve the diurnal cycle in order to determine daily maximum and minimum temperatures, which are part of the criteria for tree growth.
- Larger values of CO₂ and and artificial warming leading to much larger values of the infrared opacity were included in the greenhouse effect. The methodology for the computation of the greenhouse flux was not changed and was as described in the Baseline model.
- Modification of the subsurface flux calculation in the diurnal application of the model to account for the dependency of the damping depth on period. Instead of a damping depth corresponding to the Martian year in the diurnal model we use a damping depth corresponding to the Martian sol.

The methodology of computations assumes two main steps (Fig 1):

(i) calculation of daily average energy balance Martian model for each sol (Martian day) and for each cell;
(ii) (ii) calculation of diurnal energy balance Martian model for every sol and cell.

Both models are based on the equation for energy balance.

The model initializes with a total exchangeable CO₂ pressure, including atmospheric CO₂, polar cap CO₂, and surface frost. As the simulation progresses through seasonal cycles, CO₂ condenses or sublimates based on the balance between atmospheric pressure and local saturation pressure. The CO₂ mass flux between phases adjusts dynamically, influencing planetary pressure and thermal balance. The model determines the insolation in every cell for a specific sol. The insolation is computed using a standard orbital equation incorporating Mars’ eccentricity, axial tilt, and the areocentric longitude. The result is the solar insolation at the top of the Martian atmosphere, influencing surface energy dynamics. Latent heat is computed assuming the relation between atmospheric and local saturation pressure. The condensation takes place when atmospheric CO₂ pressure exceeds saturation pressure, leading to latent heat release and pressure reduction. On the contrary, sublimation occurs when condensed CO₂ is present, and the overlying atmospheric CO₂ pressure is below saturation pressure, cooling the cell and increasing atmospheric CO₂ mass. The iterative approach ensures that the CO₂ flux stabilizes within model constraints. Heat transport is modelled using a convection-diffusion equation, incorporating the Laplacian operator over cells in the Goldberg polygonal representation of a Mars surface. Hadley cell-driven heat transport redistributes energy from equatorial to polar regions. The model estimates heat flux convergence using zonal wind and temperature gradients, parameterized based on NASA Ames Research Center GCM model. A Fourier series approximation is used to represent seasonal variations. Heat conduction into the subsurface is computed based on thermal conductivity, depth-dependent temperature variation, and the Carslaw-Jaeger solution for periodic surface temperature fluctuations. The damping depth is set according to annual temperature penetration depth estimates. The model calculates the downward infrared flux based on surface temperature and infrared opacity, primarily influenced by CO₂ and atmospheric dust. This summarized methodology ensures computational efficiency while maintaining physical accuracy in Martian climate modeling.

The diurnal energy balance Martian model is computed using the results of the average daily energy balance Martian model for each sol separately. It takes into consideration average daily surface temperature, subsurface temperature, atmospheric pressure, greenhouse flux, radiation flux, eddy diffusion, Hadley diffusion, latent flux, frozen temp, season, and coordinates. Moreover, it takes into account the sun’s position over the horizon, calculating the insolation for every half an hour (48 times a sol), assuming sunrise and sunset, day and night. At night, the insolation is zero. The model calculates the exact energy from insolation, and exact exchange of energy with subsurface. Remaining energy components are taken as constants calculated in the baseline model [64].

## Results of the temperature calculation

**Fig 2:**
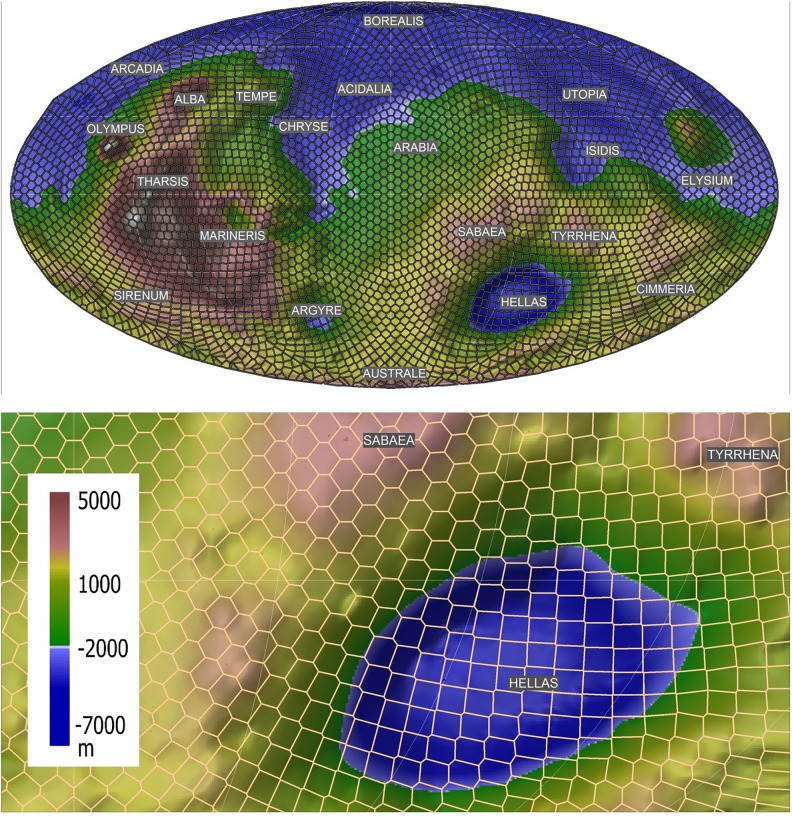
Mars topography (MOLA) with 4002 Goldberg polygons marked (top). Hellas region zoomed in (bottom). Polygons are ~ 190 km across. The shape distortion is a result of the projection from a sphere to a plane. Topographic data from the Mars Orbiter Laser Altimeter (MOLA) onboard NASA’s Mars Global Surveyor mission (public domain). Map derived and processed by the authors.

We have used a surface energy balance model for Mars based on representing the surface of Mars with a Goldberg polyhedral of 4002 cells (12 pentagons and 3990 hexagons) with each cell being about 190 km across (Fig 2). The model is described in detail in [64] and is only briefly summarized here.

The energy balance equation is applied to each cell. In addition to the radiation terms, the surface energy balance includes the diffusive exchange of heat between cells, CO₂ condensation and evaporation, heat exchange with the subsurface, and the transport of heat by eddy diffusion and by the large scale atmospheric circulation [64].

We calibrate the model parameters by comparing them to the Viking Lander temperature and pressure datasets, and by comparison to Mars GCMs, specifically the MCD model [63], and the NASA-Ames MGCM. Our model has high spatial resolution but is still computationally efficient and can be used to simulate a variety of processes on Mars, both at present and in past/future epochs. Here, we use the baseline model to investigate the greenhouse effect caused by an increase in CO₂ together with artificial greenhouse warming, and we calculate the daily average temperature for each cell for each sol using the energy balance framework described by Olszewski et al. [64]. The model is also used calculate the diurnal variation of the temperature for cells and sols of interest.

**Fig 3:**
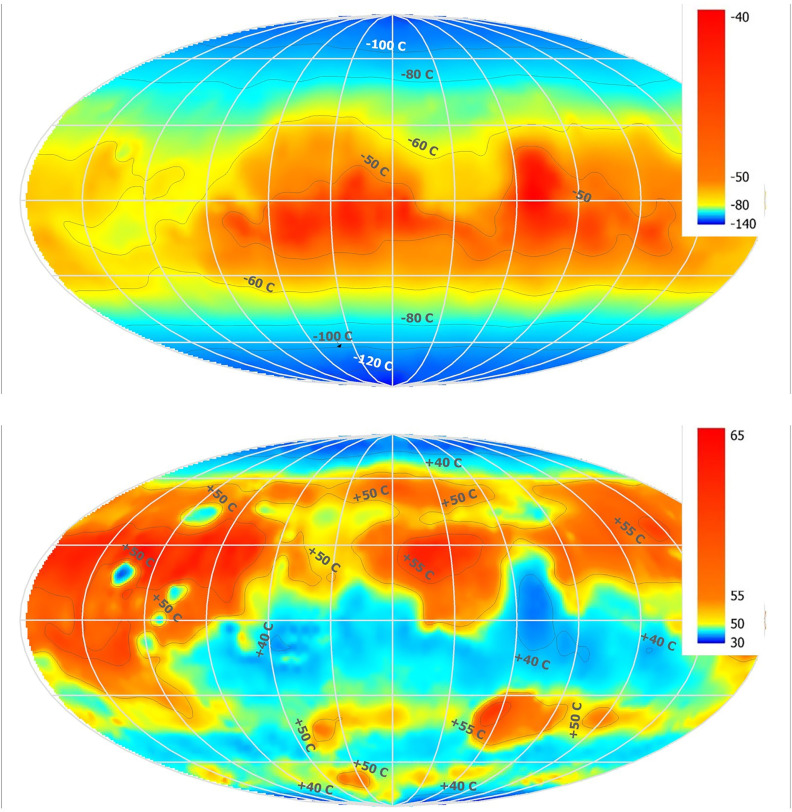
Average annual temperature on present Mars (based on MCD model [63]). (top) Average annual temperature increase on Mars in our model (atmospheric pressure 100 hPa and added grey opacity of 0.39) relative to the MCD model. (bottom).

**Fig 4:**
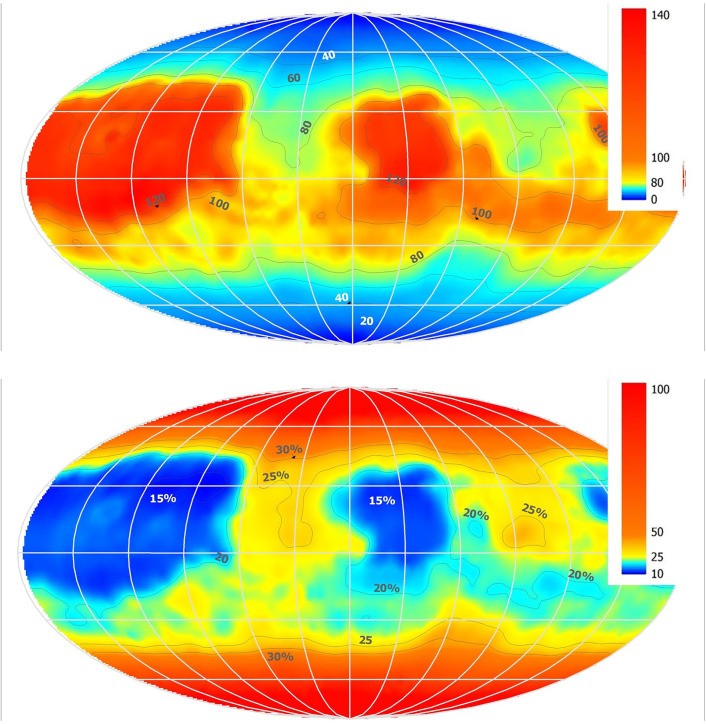
Average daily temperature spread on present Mars (based on MCD model [63]). (top) Average daily temperature spread on Mars in our model shows annually averaged daily temperature range in the 100 hPa model with 0.39 optical depths expressed as the percentage of the range in the MCD model (bottom).

We present results for an assumed CO₂ surface pressure of 100 hPa, 17 times the present value, and one which, as discussed above, is a total pressure known to support plant growth and is in the low range of estimates of the available CO₂. The results obtained with our model for an atmospheric pressure of 100 hPa and added grey opacity of 0.39 are shown in Fig 3. Fig 3 (top) is the annual average temperature on Mars as computed from the MCD model. Fig 3 (bottom) shows the increase in the annual average temperature above the MCD results in Fig 3 for 100 hPa of CO₂ and added grey opacity of 0.39. The resolution of the results in Fig 3 is limited by the spatial resolution of the MCD model and is too coarse to accurately determine the locations of tree growth. The increase in average annual temperature is uneven and particularly high in the Hellas, Tharsis, Arabia, Elysium and Utopia regions (Fig 3 bottom). Fig 4 (top) shows the annually averaged daily temperature range (daily maximum – daily minimum) on Mars from the MCD model. Fig 4 (bottom) shows the annually averaged daily temperature range in the 100 hPa model with 0.39 optical depths expressed as the a percentage of the range in the MCD model (Fig 4 top). In Tharsis and Arabia, the average daily temperature differentials for our model are about 15% of the values for the MCD model. In the polar regions, the percentage is about 50% of the baseline value from the MCD model (Fig 4 bottom). These results show that, as expected, a thicker atmosphere with greenhouse gases on Mars warms the planet (Fig 3 bottom) and reduces the range of daily temperature swing (Fig 4 bottom).

**Fig 5:**
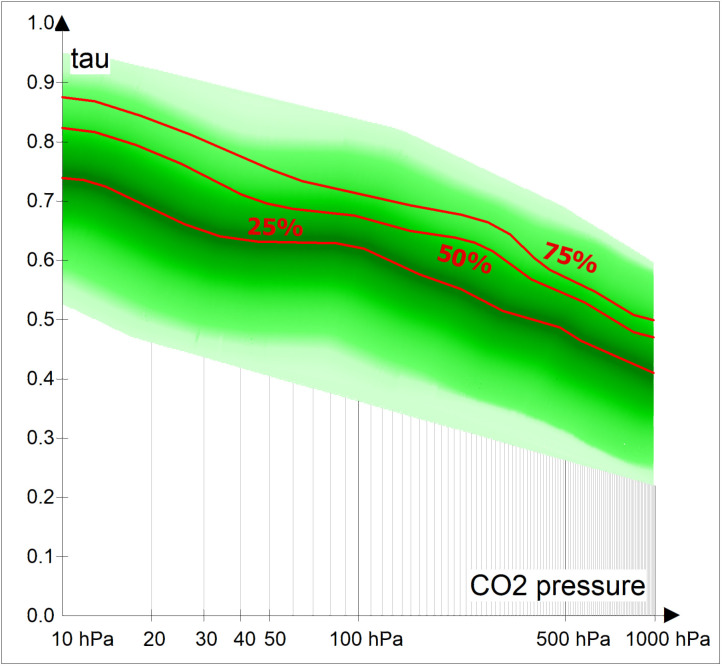
Boundary conditions of CO₂ and grey opacity values for tree growth on Mars. Below the green zone conditions are too cold for tree growth. Within the green zone the darker the green color, the greater the proportion of Mars’ surface that allows plant growth (the darkest green corresponds to ~75% of the surface being suitable for tree growth. Above the dark green zone increase in CO₂ or greenhouse opacity reduce the fraction of the planet suitable for tree growth as a result of high temperatures. The red lines indicate what percentage of the surface is too hot to allow plant growth.

For the 100 hPa CO₂ atmosphere we find that temperatures suitable for trees to grow can occur when the added, artificial, greenhouse thermal infrared grey opacity is ~ 0.39 optical depths. We can explore the warming effect of additional CO₂, setting aside for now the toxicity of high CO₂, and consider the temperature limits of tree growth [65,66]. If the CO₂ pressure is increased to 1000 hPa, the threshold grey opacity value is ~ 0.22, while for a pressure of 10 hPa, this value is ~ 0.5. We show in Fig 5 the range of conditions that would allow tree growth based on temperatures alone (excluding O_2_ and CO₂ limits, and water considerations).

It should be noted that the conditions (i – iv) defined above that allow tree growth, based on thermal requirements only, are limited not only by the minimum (ii), but also by the maximum (iv) temperature. Maximum daily temperature above 40°C occurring for 5 sols in row is assumed to prevent trees from growing.

**Fig 6:**
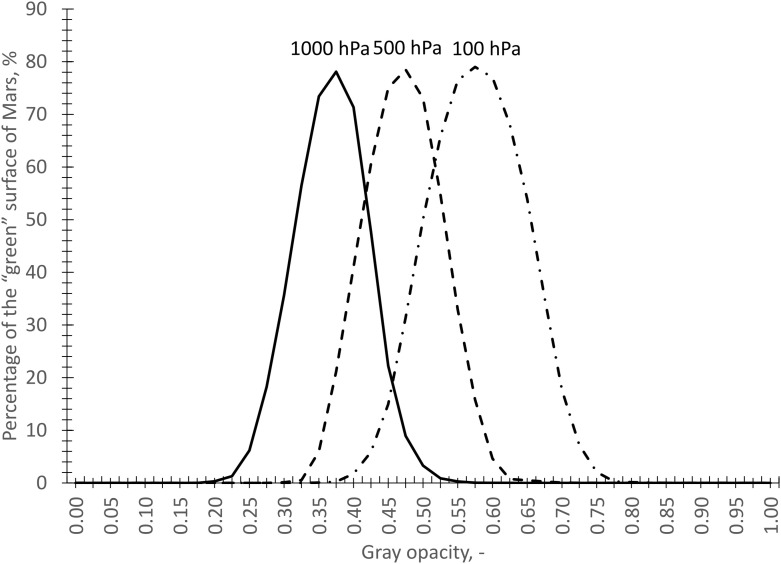
Percentage of Mars surface area allowing tree growth depending on atmospheric CO₂ pressure and grey opacity values.

**Fig 7:**
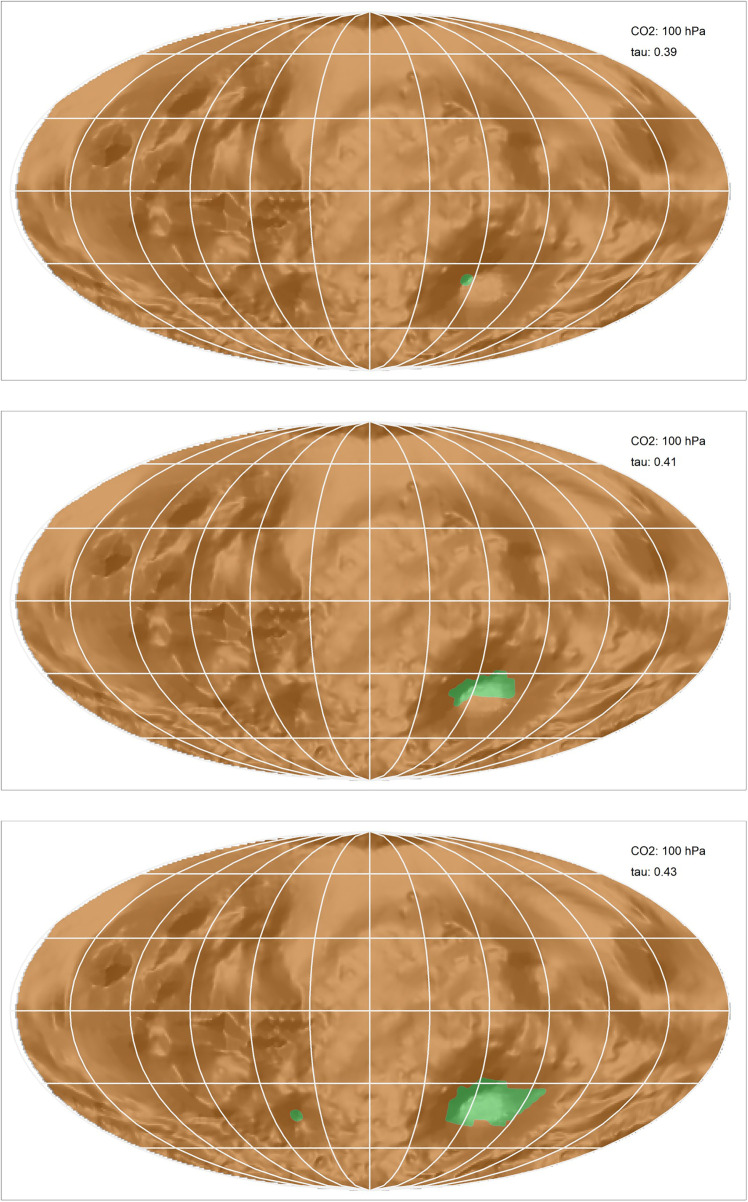
Model calculation for different values of atmospheric pressure and different values of the grey opacity parameter. Color scheme as in Fig 5. Topographic data from the Mars Orbiter Laser Altimeter (MOLA) onboard NASA’s Mars Global Surveyor mission (public domain). Map derived and processed by the authors.

These conditions determine that for an increasing value of grey opacity, the percentage of Mars’ area on which trees could grow initially increases (darker shading on Fig 5), and then decrease after reaching a threshold value of about 75%. This is illustrated in Fig 5 where the red lines show the percentage of the planet’s surface that is too hot to provide conditions for tree growth. For an CO₂ atmospheric pressure of 100, 500, and 1000 hPa, this phenomenon is also illustrated in Fig 6, which reinforces that as we go to higher pressures, a smaller area on Mars can allow tree growth Fig 7.

**Fig 8:**
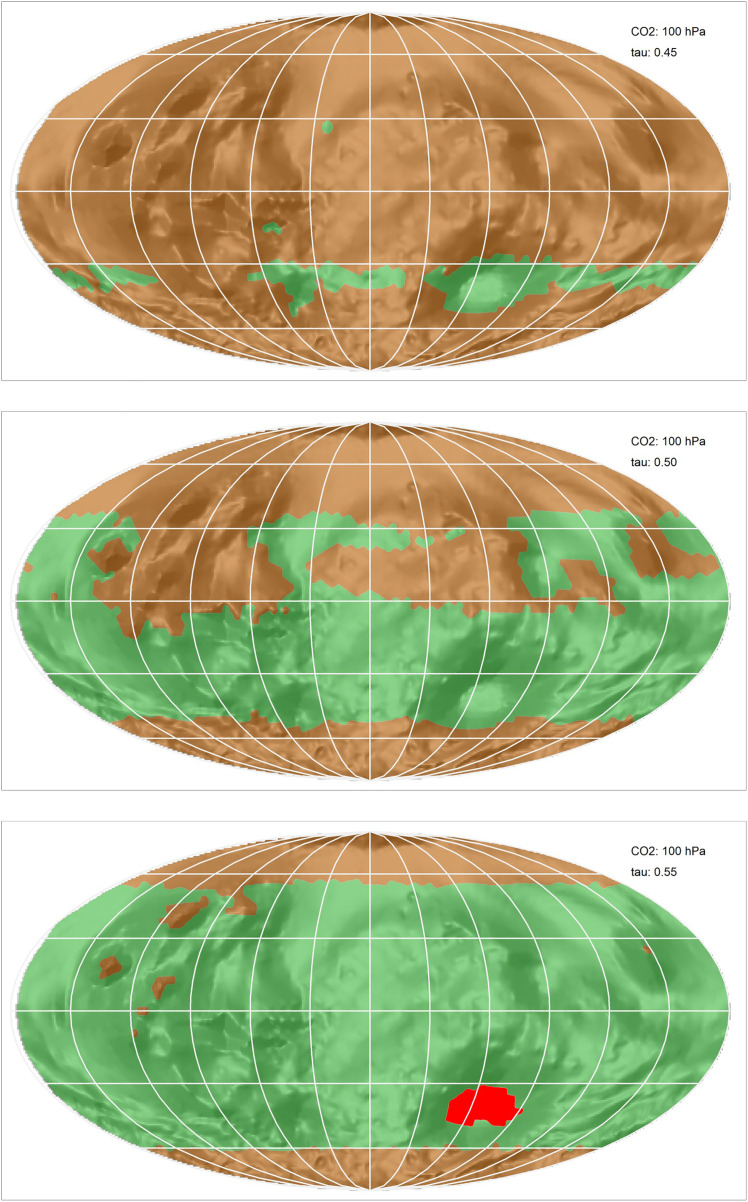
Model calculations for a CO₂ atmospheric pressure of 100 hPa and increasing values of the grey opacity parameter. Green shading indicates areas that allow tree growth, while red shading shows regions that are too hot). Topographic data from the Mars Orbiter Laser Altimeter (MOLA) onboard NASA’s Mars Global Surveyor mission (public domain). Map derived and processed by the authors.

**Fig 9:**
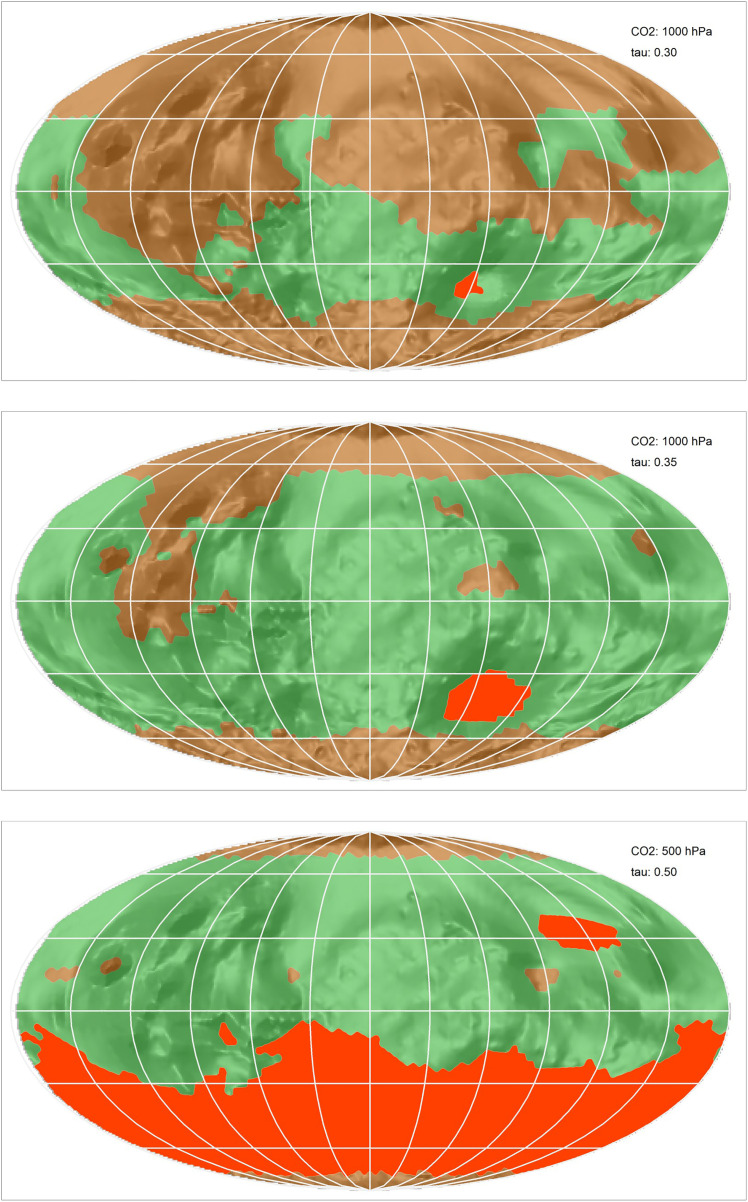
Model calculation for different values of atmospheric pressure and different values of the grey opacity parameter. Color scheme as in Fig 5. Topographic data from the Mars Orbiter Laser Altimeter (MOLA) onboard NASA’s Mars Global Surveyor mission (public domain). Map derived and processed by the authors.

Surprisingly, the conditions that allow for tree growth do not occur first within the tropics (±25° latitude) but in the Hellas Basin region (Figs. 2). A further increase in the greenhouse effect expands the area suitable for plant growth in the southern hemisphere. For a value of grey opacity = 0.55, the maximum daily temperature in the Hellas area regularly exceeds 40°C during the summer, making the area too hot for trees to grow. This phenomenon intensifies not only as the value of the thermal infrared grey opacity increases (Fig 8), but also when higher atmospheric pressures are assumed in the model. For an atmosphere composed of CO₂ at a pressure of 1000 hPa, the process of “overheating” Hellas already occurs for a value of grey opacity of 0.30. Similarly, for an atmospheric pressure of 500 hPa and grey opacity of 0.50, almost the entire southern hemisphere of the planet is too hot in the summer to allow tree growth (Fig 8, red shaded areas) Fig 9.

## Discussion and conclusions

Our results should be interpreted strictly as an analysis of surface temperature and pressure conditions under deliberately simplified atmospheric assumptions, rather than as a full assessment of biological feasibility or a realistic terraforming pathway. The model isolates the thermal response of the surface–atmosphere system to changes in total CO₂ pressure and an imposed grey infrared opacity, and does not explicitly represent the coupled water cycle, detailed atmospheric dynamics, photochemistry, or the radiation and geochemical environments that would confront real organisms. Consequently, the “growth regions” identified here mark locations and parameter combinations where thermal pre‑conditions for tree growth, as inferred from terrestrial treeline studies, could in principle be satisfied, but they do not by themselves guarantee that trees could survive or that a self‑sustaining biosphere could be established. Accordingly, the “growth regions” identified in our maps should be read as thermal candidates only. They indicate where temperature and pressure conditions could, in principle, be compatible with tree‑level physiology, but their atmospheric and biogeochemical feasibility – particularly the simultaneous satisfaction of total pressure, O₂ supply, and non‑toxic CO₂ - remains to be tested using more self‑consistent atmospheric and photochemical models.

Our findings should therefore be regarded as a conceptual, temperature‑focused benchmark that constrains one necessary aspect of potential tree viability on a terraformed Mars, to be complemented in future work by more comprehensive treatments of hydrology, atmospheric composition, radiation, soils, and biology.

Within this modelling structure, the maps and percentage values presented in this work should be interpreted strictly as indicating where and when thermal conditions alone would cease to be the dominant limitation for terrestrial tree growth. They identify spatio‑temporal thermal windows under simplified atmospheric assumptions, but they do not imply that these regions are fully habitable or that other key constraints – such as the availability and state of water, soil development, radiation shielding, or self‑consistent atmospheric composition – are automatically satisfied. In any realistic terraforming scenario, these additional factors would need to be provided or engineered by other means before complex plant life could persist.

Our results complement previous conceptual climate engineering studies for Mars by quantitatively linking surface temperature fields to empirical treeline criteria derived from high‑elevation and high‑latitude forests on Earth [33–35]. It is important to stress that these treeline‑derived thresholds are calibrated in terrestrial mountain environments with active hydrology, mature soils, and abundant O₂, and are used here only as proxy thermal limits in a conceptual exercise. As a consequence, the locations and extents of the “thermal windows” we identify should be viewed as indicative of where tree‑level physiology might in principle be supported, rather than as exact predictions for any specific species under Martian conditions. In the broader context of planetary science and astrobiology, this work provides a benchmark for the thermal conditions required for complex multicellular plant life, which is distinct from the lower thresholds relevant for microbial habitability or for spectroscopic biosignatures in exoplanetary atmospheres. A more complete assessment of tree viability on Mars would require coupling our energy balance framework to photochemical models of O₂ and CO₂, detailed water cycle simulations under thick atmospheres, and experimental studies of plant physiology under combined low‑pressure, high‑CO₂ and low‑O₂ conditions.

This work should therefore be regarded as a conceptual exercise that isolates the effects of atmospheric pressure and surface temperature on the potential for tree growth, rather than as a comprehensive assessment of Martian terraforming feasibility, which would also need to account for radiation, regolith geochemistry, water availability, photochemistry, and broader astrobiological constraints.

These considerations imply that the “growth regions” identified in our maps represent thermal candidates only, whose biological relevance is contingent on simultaneously satisfying stringent constraints on atmospheric composition. The locations and extents of the thermal windows are determined by total pressure and grey infrared opacity, whereas real trees would additionally require that the CO₂ partial pressure remain below ~10 hPa (ideally ~1 hPa), that sufficient O₂ be available for respiration, and that other environmental factors (water, soils, radiation) be favorable. In other words, our pure‑CO₂ simulations explore where and when temperature would cease to be the primary limiting factor if a suitable N₂–CO₂–O₂ mixture and supporting environment could be engineered by other means. A full assessment of tree or forest viability on Mars must therefore couple our thermal constraints to self‑consistent atmospheric, hydrological, and biological models; here we provide only the first, thermal step in this chain.

**Fig 10:**
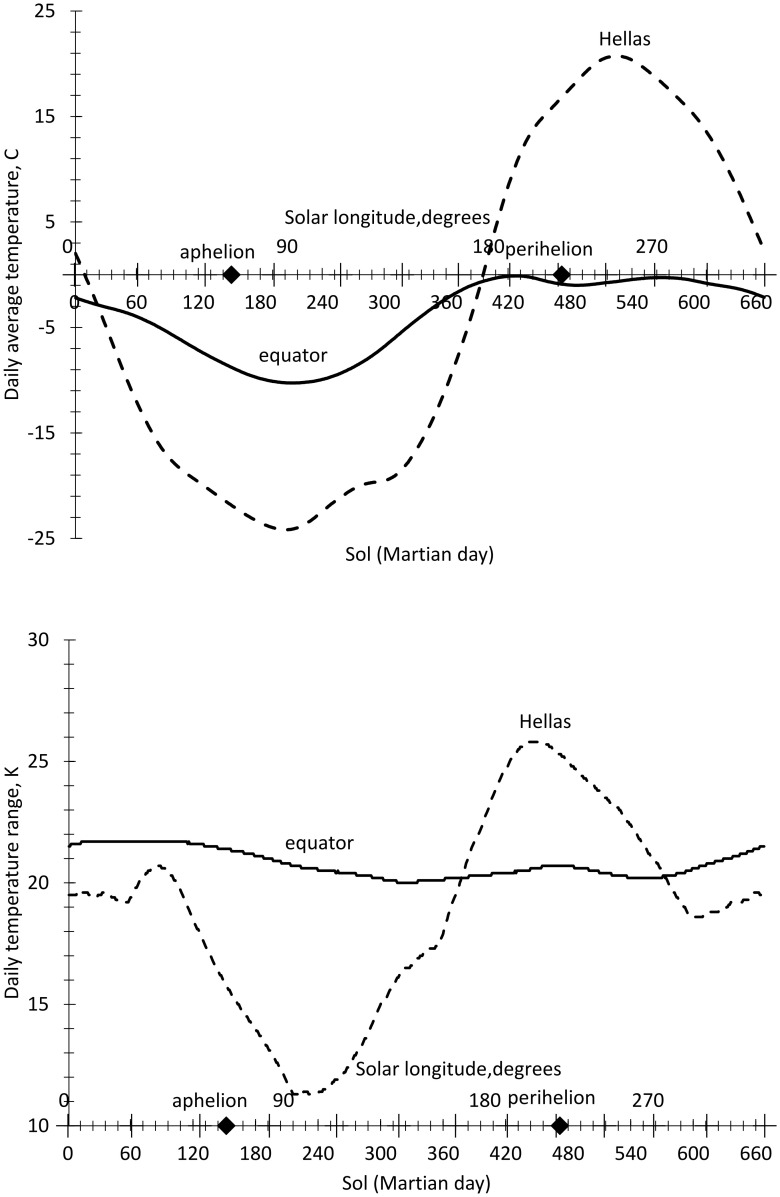
Comparison of daily mean temperature values (top) and daily temperature spread for Hellas and the equator (bottom).

Our surface energy balance model of Mars suggests that suitable temperature conditions for the first tree on that planet could appear when the mean surface temperature is 6°C with a surface CO₂ pressure of 100 hPa plus artificial greenhouse opacity of ~0.4. In particular, while the 100 hPa case lies within the low end of current estimates for accessible CO₂, the 500–1000 hPa scenarios should be regarded as purely illustrative high‑pressure extensions rather than realistic terraforming targets. On Earth, the highest elevation treelines are primarily found in the tropics – as modulated by the location of the thermal equator [67]. Thus, it may be expected that equatorial regions of Mars would be the location of the first tree. However, due to Mars’ relatively large orbital excentricity (~0.1), the southern hemisphere, which has its summer near perihelion, has relatively warm summers. In addition, the orbital period of Mars is ~ 1.9 Earth years. Thus, the long warm southern summer provides the first growing season suitable for trees. Specifically, we find that the low elevation of the Hellas Basin allows the development of the thermal conditions favorable for tree growth. Comparing the average daily value of surface temperature, it can be seen that our model with an atmospheric pressure of 100 hPa and an added grey greenhouse opacity of 0.39 predicts significantly warmer days during the growing season for the Hellas region than at the equator at the same longitude (Fig 10).

For complex multicellular organisms such as trees, however, water availability is likely an equally important or even more stringent limiting factor than temperature. Our model does not include an explicit hydrological cycle and therefore cannot predict where liquid water would be stable at the surface or in the shallow subsurface under the thick‑atmosphere scenarios considered here. Early‑Mars and high‑obliquity GCM studies [53,60,61] indicate that increasing atmospheric pressure generally enhances the efficiency of water transport away from the poles, but that achieving long‑lived surface liquid water still requires additional warming and remains non‑trivial. In this context, the regions we identify should be regarded as zones where temperature would no longer be the primary constraint if adequate and reliably accessible water could be supplied by other means, rather than as areas where hydrological feasibility is guaranteed.

Our results show that because of the large topographic relief on Mars and the high eccentricity of its orbit, high spatial resolution is needed to determine the locations where thermal conditions would allow for tree growth on a warming Mars. We find that initially, Mars is too cold everywhere for tree growth. As the greenhouse effect increases, conditions in small regions become suitable for tree growth, provided the direct effects of the adverse oxygen and CO₂ effects on trees are resolved. Further increases in the greenhouse effect initially expand the area suitable for trees but eventually result in conditions at some locations that are too hot. The fraction of Mars that could, in our model, thermally be made suitable for tree growth peaks at ~80% of the surface; this purely temperature‑ and pressure‑based estimate, which deliberately ignores constraints such as water availability, soil properties, radiation, and atmospheric composition, should not be interpreted as directly comparable to the ~ 30% of Earth’s land surface currently covered by forests.

Taken together, our results therefore define a thermal pre‑condition – or “thermal window” – for potential tree growth on Mars, rather than a full habitability criterion, and should be viewed as a first step to be combined in future work with explicit treatments of hydrology, atmospheric composition, radiation, soils, and biology.

In this study, we focused on the temperature increase due to higher CO₂ pressure plus additional artificial greenhouse warming. We assumed an atmosphere at the lowest pressure that plants have been shown to grow (100 hPa). For the purposes of the thermal calculation, we assumed pure CO₂. However, it may be the case that the necessary level of CO₂ is determined not by temperature but by the competing upper limit set the toxicity of CO₂ and the lower limit set by the photochemical production of sufficient O_2_ for a plant to thrive on Mars. Both of which are areas for future research. Future work should therefore couple this type of high‑resolution surface energy balance framework with (i) 3‑D climate and hydrological models that explicitly simulate the water cycle under thick CO₂ atmospheres, (ii) photochemical models of O₂ and CO₂ to constrain atmospheric composition, and (iii) laboratory and analogue‑site experiments on plant and microbiome performance under Mars‑relevant pressure, gas composition, temperature, and radiation regimes, thereby linking our conceptual thresholds more directly to ongoing astrobiological and planetary science investigations.

In future work, the spatio‑temporal thermal windows identified here could be explored and communicated using immersive visualization tools for planetary data (e.g., [68]), which may help both scientists and the broader public to better understand the spatial structure of potential terraforming scenarios.
